# Neutrophil extracellular traps and dsDNA predict outcomes among patients with ST-elevation myocardial infarction

**DOI:** 10.1038/s41598-019-47853-7

**Published:** 2019-08-12

**Authors:** Jing Liu, Dandan Yang, Xiqiang Wang, Zhonghai Zhu, Tingzhong Wang, Aiqun Ma, Ping Liu

**Affiliations:** 1 Department of Cardiovascular Medicine, First Affiliated Hospital of Xi’an Jiaotong University, Xi’an, P.R. China; 2 Cardiovascular Department, the Second Affiliated Hospital, Zhejiang University School of Medicine, Hangzhou, 310009 Zhejiang China; 3 Department of Epidemiology and biostatistics, School of Public Health, Xi’an Jiaotong University Health Science Center, Xi’an, P.R. China; 4 Key Laboratory of Molecular Cardiology, Shaanxi Province, P.R. China; 5 Key Laboratory of Environment and Genes Related to Diseases (Xi’an Jiaotong University), Ministry of Education, Xi’an, P.R. China

**Keywords:** Atherosclerosis, Acute coronary syndromes, Interventional cardiology

## Abstract

Neutrophil extracellular traps (NETs) which have a potential role in noninfectious diseases, may play an important role in patients with acute coronary syndrome. The goal of this study was to investigate the association of NETs and in-hospital major adverse cardiac events among patients with ST-segment elevation myocardial infarction (STEMI). Using immunofluorescence staining, ELISA, and fluorescent enzyme standard instrument, we assessed NETs and NETs-related factors. Multivariate analyses were performed after univariate analyses to investigate which variables were independently associated with major adverse cardiac events. Compared with peripheral arteries, we observed neutrophils obtained from infarct-related artery (IRA) releasing NETs. The dsDNA levels, NET-specific marker myeloperoxidase/deoxyribonucleic acid (MPO/DNA) complexes and NETs-related factor tissue factor were significantly higher in coronary plasma samples. Multivariate analysis that white cell counts and coronary dsDNA were independently associated with in-hospital major adverse cardiac events. ROC curve for coronary dsDNA showed sensitivity of 78.0% and specificity of 53% for the cut-off value of 0.39ug/ml. Conclusion, these results provide evidences indicating NETs were associated with STIM, and occurrence of adverse cardiac events.

## Introduction

Atherosclerotic plaque disruption and subsequent intraluminal thrombus formation are the primary pathological hallmark of acute myocardial infarction. Despite modern advances in both pharmacological and interventional therapy, atherothrombosis remains one of the most significant clinical burdens worldwide^1^. Therefore, precise pathological mechanisms of acute coronary occlusion and the factors that influence complications and prognosis must be explored.

Neutrophils, the front-line defense cells against microbes^2^, have another distinct microbicidal function in relation to NETs^3,4^. NETs are web-like structures, comprising decondensed chromatin coated with granular proteins such as MPO and neutrophil elastase (NE)^5,6^. Recent evidence shows that NETs may have a potential role in noninfectious diseases, including, but not limited to, atherosclerosis^7^.

In patients with severe coronary atherosclerosis, plasma NETs-related structures such as dsDNA, nucleosomes, and MPO/DNA complexes are elevated. Meanwhile, the level of nucleosomes is associated with the risk of coronary stenosis, whereas MPO/DNA complexes can predict major adverse outcomes^8^. In the blood of infarcted coronary artery, neutrophils aggregate in large numbers. However, it is not clear whether infarct-related coronary neutrophil released NETs or NET-related factors and their related structures are associated with adverse outcome in patients with acute STEMI.

Mounting evidence has implicated NETs in sterile inflammation^9–12^. We hypothesized that neutrophils/NETs may play an important role in STEMI. Therefore, we assessed the IRA structures of NETs and their related factors in STEMI patients. We also evaluated the relationship between the occurrence of NET-related markers and in-hospital MACEs.

## Results

### Patient characteristics

We enrolled 83 consecutive patients in this study. Each patient was enrolled within 12 hours of the onset of clinical signs and had STEMI with TIMI flow 0 before emergent PCI. Characteristics of patients are listed in Table 1. Approximately 78.9% of the patients were men, the mean age was 59 ± 1years, and conventional risk factors were similar to those of recently published ST-elevation acute coronary syndrome (STE-ACS) trials. Two study participants died in hospital.

**Table 1:** Baseline of Patient characteristics.

Age, y	59 (59 ± 1)
Sex, female, n (%)	24 (17.9%)
Ever smokers, n (%)	46 (57.5%)
Diabetes mellitus, n (%)	14 (17.5%)
History of hypertension, n (%)	33 (41.3%)
**Culprit vessel, n (%)**	
LAD	39 (48.8%)
CX	7 (8.8%)
RCA	29 (36.3%)
LM	3 (3.8%)
Others	2 (2.5%)
CRP, nmol/L (<4.8)	8.8 (8.8 ± 2.3)
CK, U/L (<200)	1799 (1799 ± 310)
Peak of CK	4624 (4624 ± 271)
CK-MB, U/L (<24)	133.4 (133.4 ± 18.7)
Peak of CK-MB	379 (379 ± 198)
TnT, μg/L (0–0.03)	1.40 (1.40 ± 0.32)
Cholesterol, mmol/L (<5.2)	4.1 (4.1 ± 0.1)
Triglycerides, mmol/L (<1.7)	1.95 (1.95 ± 0.30)
LDL, mmol/L (<4.1)	2.41 (2.41 ± 0.08)
HDL, mmol/L (>1.5)	0.98 (0.98 ± 0.02)
BNP, pg/mL (<400)	1286 (1286 ± 205)
Ejection fraction, % (55–70)	50 (50 ± 1)
Final TIMI	3.9 (3.9 ± 0.05)
Admission to PCI time	8.2 (8.2 ± 2)

Data are presented as mean ± SD, or number (percent) of patients. Normal ranges are given in parenthesis. LAD, left anterior descending artery; CX, circumflex artery; RCA, right coronary artery; LM, left main coronary artery; CRP, C-reactive protein; CK, creatine phosphokinase; CK-MB, creatine phosphokinase isoform MB; TnT, troponinT; LDL, low density lipoprotein cholesterol; HDL, high density lipoprotein cholesterol; BNP, brain natriuretic peptide; TIMI, Thrombolysis in myocardial infarction; PCI, Percutaneous coronary Intervention.

### Neutrophils from infarct-related coronary arteries release NETs structures and NETs-related factors

**Figure 1:**
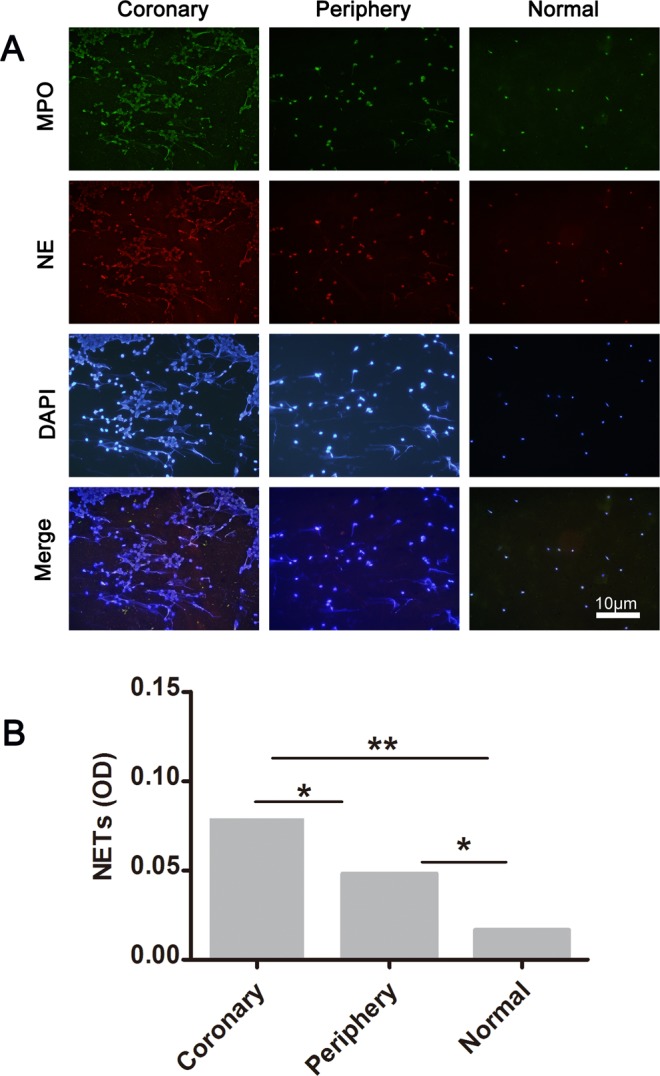
Neutrophils from infarct-related artery and peripheral artery blood of same patients with STEMI form NETs. (**A**) Immunostaining for NETs obtained from infarct-related artery, peripheral artery blood (n = 36), and rare phenomenon was observed in any healthy control individual(n = 5), and the proportion of neutrophils with NETs was significantly higher in STEMI than healthy individual. (**B**) Integrated optical density(OD) of NETs were measured using ImageJ software, and NETs OD per area in coronary artery was higher than radial artery and control individual. Accordingly, NETs OD per area in radial artery was higher than control individual. Green: MPO; red: NE; blue: nucleus labeled with DAPI. One representative out of 36A independent experiments is shown in A. Original magnification: A × 200, scale bar in A, 10 μm, figures demonstrate as mean Optical Density (OD) for (**B**). **P < 0.01, *P < 0.05.

We observed *in vivo* neutrophils isolated from IRA blood samples (n = 36) forming NETs and significant differences were observed compared with peripheral arteries from the same patients (n = 36) and control individuals (controls, n = 5), as demonstrated by microscopy immunofluorescence (Fig. 1A,B).

**Figure 2:**
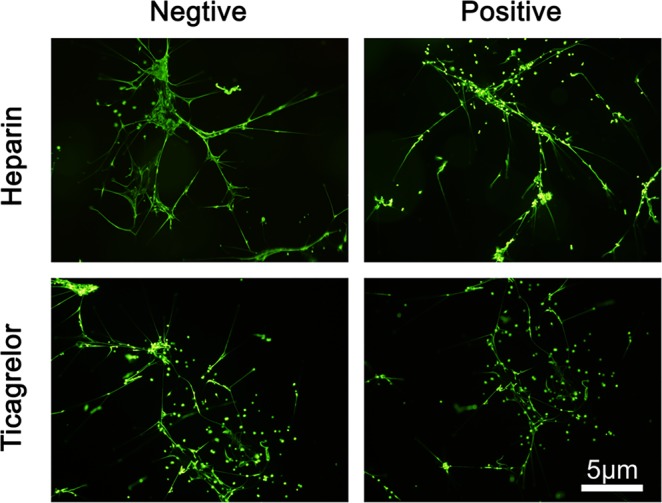
NETs in infarct-related coronary arteries were not sensitive to drugs, such as heparin or ticagrelor. Using heparin or ticagrelor treatment, do not reduced the proportion of NETs accumulating in infarct-related arteries. One representative out of 10 (Fig. 3) independent experiments is shown. Original magnification: A × 400, scale bar in C, 5 μm. Green: NETs.

Accordingly, *in vitro*, we also looking for mechanisms that antagonize NETs, and, we used heparin which may influence the activation of neutrophil forming NETs to process the NETs structures. This was ineffective, as was tirofiban (Fig. 2).

**Figure 3:**
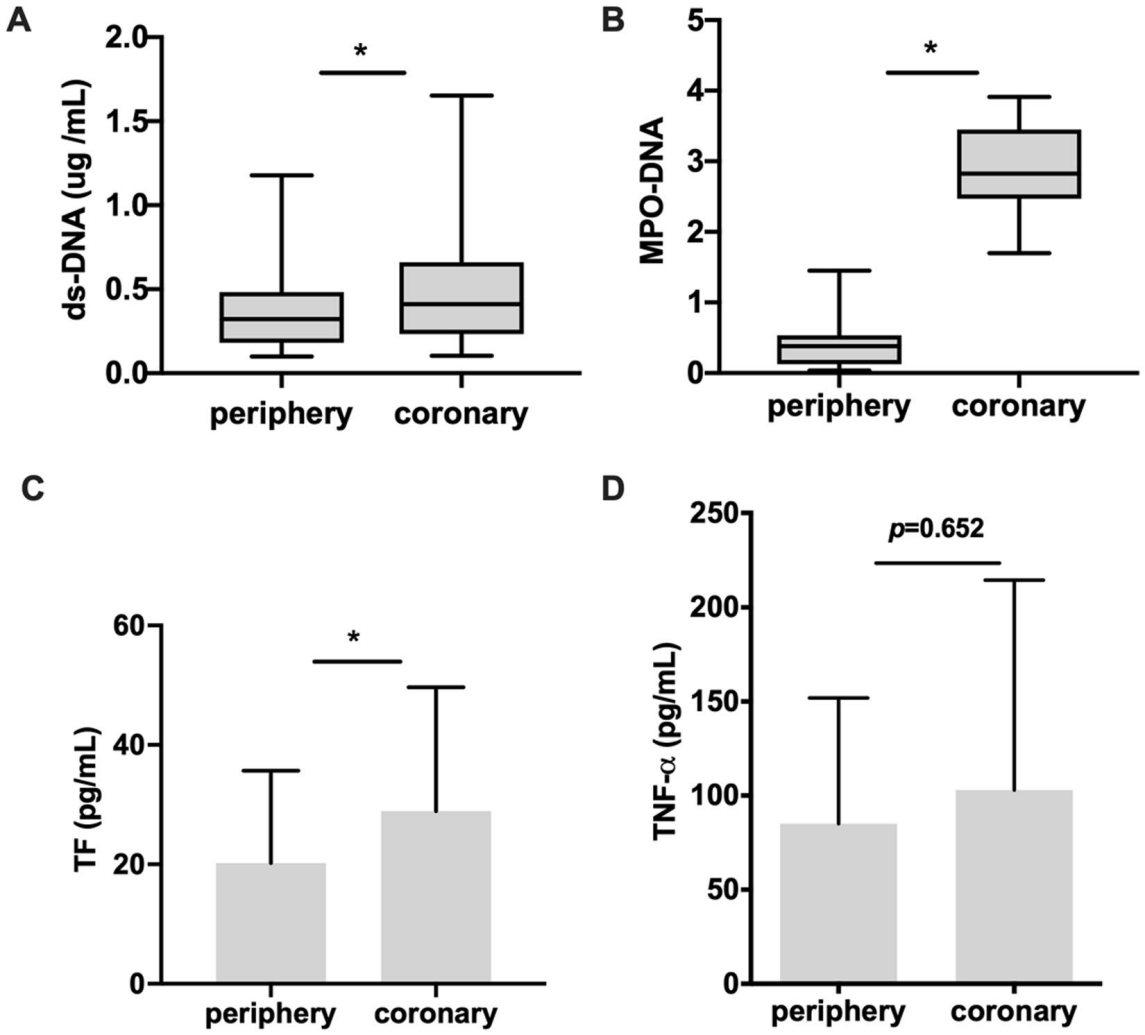
Surrogate Markers of NETs and NETs-related factors in infarct-related artery were higher than peripheral artery blood (**A**) ds-DNA (**B**) MPO/DNA and (**C**) TF in plasma samples from coronary arteries increased compared with peripheral arteries. (**D**) There was no significant difference for TNF-α between coronary and periphery blood.All figures demonstrate as mean ± SD, *P < 0.05.

Then the extracellular DNA (mean 0.41 *vs*.0.31, *p* = 0.038), NET-specific marker MPO/DNA complexes (mean 0.44 *vs*. 0.28, *p* = 0.021) and NETs-related TF (mean 22.0 *vs*. 15.7, *p* = 0.048) were significantly higher in plasma samples obtained from IRA compared with peripheral arteries (Fig. 3A–C). However, there was no significant difference for TNF-α between coronary and periphery blood (mean 86 *vs*.78, *p* = 0.652) **(**Fig. 3D).

### High baseline levels of coronary dsDNA are significantly associated with the occurrence of in-hospital adverse events

During hospitalization, 23(27.7%) patients suffered adverse events. (Table 2).

**Table 2:** Type of in-hospital Major adverse cardiac events.

Type of adverse events	Patients
Cardiogenic shock	n = 14
Ventricular arrhythmias	n = 6
Infarction recurrence	n = 1
Heart failure	n = 1
Cardiac deaths	n = 1
Total	n = 23

In-hospital adverse events occurred more frequently in men (72.7%), with a mean age of 59 years (59** ± **1), who were less often diabetic (9%). Significantly elevated baseline levels of coronary dsDNA, were observed in the group who suffered adverse events compared with the event-free group (mean 0.46ug/ml in non-MACEs vs. 0.70ug/mL in MACEs; *p* = *0.002*). In addition, in-hospital MACEs were associated with following factors: white cell counts, mononuclear cells, troponin T, systolic blood pressure, coronary dsDNA (*p* < *0.05)*. (Table 3).

**Table 3:** Description of patient association of different variables with in-hospital MACEs.

	Full Patients (n=83)	In Hospital MACEs		*p* value
		MACEs (n = 23)	NonMACEs(n = 60)	
Age(years)	59 (59 ± 1)	61 (61 ± 3)	59 (59 ± 1)	0.407
Female sex	16 (19.3%)	7 (30.4%)	9 (15%)	0.113
**Culprit vessel, n (%)**				
LAD	39 (48.8%)	12 (52.2%)	29 (48.3%)	0.902
CX	7 (8.8%)	1 (4.3%)	7 (11.7%)	0.874
RCA	29 (36.3%)	9 (39.1%)	20 (33.3%)	0.315
LM	3 (3.8%)	1 (4.3%)	2 (3.3%)	0.935
Others	2 (2.5%)	0	2 (3.3%)	0.999
White cell counts	11.8 (11.8 ± 0.4)	13.8 (13.8 ± 0.9)	11.0 (11.0 ± 0.4)	0.011
Mononuclear cells counts	0.5 (0.5 ± 0.07)	0.7 (0.7 ± 0.02)	0.4 (0.4 ± 0.02)	0.008
CK-MB, U/L (<24)	133.4 (3.4 ± 18.7)	161 (161 ± 36)	122 (122 ± 21)	0.711
TnT, μg/L (0–0.03)	1.40 (1.40 ± 0.32)	2.4 (2.4 ± 0.7)	1.0 (1.0 ± 0.3)	0.048
CK, U/L (<200)	1799 (1799 ± 310)	2366 (2366 ± 673)	1590 (1590 ± 345)	0.579
LDL, mmol/L (<4.1)	2.41 (2.41 ± 0.08)	2.4 (2.4 ± 0.1)	2.4 (2.4 ± 0.09)	0.954
HDL, mmol/L (>1.5)	0.98 (0.98 ± 0.02)	0.9 (0.9 ± 0.04)	0.9 (0.91 ± 0.02)	0.671
Triglycerides mmol/L (<1.7)	1.95 (1.95 ± 0.30)	2.1 (2.1 ± 0.4)	1.3 (1.3 ± 0.1)	0.212
BNP, pg/mL (<400)	1286 (1286 ± 205)	1718 (1718 ± 453)	1125 (1125 ± 224)	0.26
systolic blood pressure	126 (126 ± 2)	119 (119 ± 4)	128 (128 ± 2)	0.039
diastolic pressures	79 (79 ± 1)	78 (78 ± 2)	80 (80 ± 1)	0.487
Killip class III-IV	18 (21.7%)	7 (9.5%)	11 (11.6%)	0.314
Ejection fraction, % (55–70)	50 (50 ± 1)	48 (48 ± 2)	51 (51 ± 2)	0.393
**MPO/DNA**				
coronary	2.6 (26 ± 0.2)	2.8 (2.8 ± 0.1)	2.7 (2.7 ± 0.08)	0.099
periphiry	0.4 (0.4 ± 0.2)	0.4 (0.4 ± 0.1)	0.4 (0.4 ± 0.07)	0.213
**Cell free DNA (ug/mL)**				
coronary	0.53 (0.53 ± 0.44)	0.70 (0.70 ± 0.09)	0.46 (0.46 ± 0.5)	0.002
periphiry	0.38 (0.38 ± 0.37)	0.53 (0.53 ± 0.11)	0.38 (0.38 ± 0.02)	0.058

Data are presented as mean ± SD, or number (percent) of patients. Normal ranges are given in parenthesis. LAD, left anterior descending artery; CX, circumflex artery; RCA, right coronary artery; LM, left main coronary artery; CK, creatine phosphokinase; CK-MB, creatine phosphokinase isoform MB; TnT, troponinT; LDL, low density lipoprotein cholesterol; HDL, high density lipoprotein cholesterol; BNP, brain natriuretic peptide; MPO/DNA, myeloperoxidase/deoxyribonucleic acid; MACEs: major adverse cardiac events.

Inter-variable analyses showed that white blood cell was associated with mononuclear cells; the variable was excluded from the multivariate model that showed white cell counts and coronary dsDNA were independently associated with in- hospital MACEs (Table 4).

**Table 4:** Multivariable Logistic Regression Analysis for in-hospital MACEs.

Variable	OR	95% CI		*P* value
		LCI	UCI	
White cell counts	1.228	1.007	1.449	0.043
Troponin T	1.207	0.967	1.505	0.096
Systolic blood pressure	0.973	0.937	1.011	0.159
Coronary dsDNA	46.264	4.775	448.21	0.001

OR, odd ratio; CI confidence interval; LCI, low confidence interval; UCI, up confidence interval; MACEs: major adverse cardiac events.

**Figure 4:**
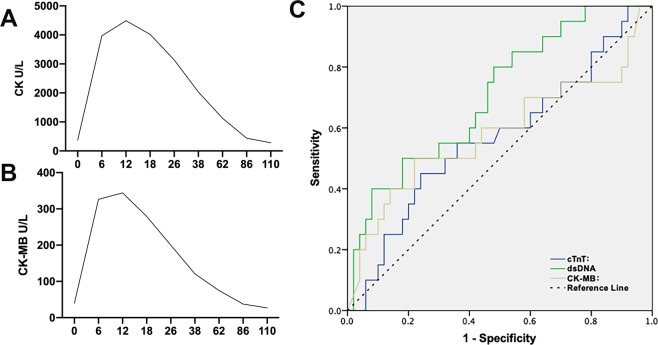
Time-concentration curves of cardiac biomarkers level and ROC curves analysis for prediction MACEs time-concentration curve of CK and (**B**) CK-MB: the curves for CK, CK-MB reached peak levels by 6 to 12 h, and decreased steadily thereafter. (**C**) The AUC of troponin T, CK-MB and dsDNA were 0.68, 0.57 and 0.72 respectively. AUC: area under curve; ROC: Receiver operating characteristic; MACEs: major adverse cardiac events.

Meanwhile, time-concentration curves and their changes for the plasma levels of total CK, CK-MB from time of enrollment to 110 h have been displayed (Fig. 4A,B), which was commonly used in evaluating the degree of myocardial necrosis in clinical practice and associated with adverse clinical outcomes^13–15^. The curves for CK, CK-MB reached peak levels by 6 to 12 h, and decreased steadily thereafter. The concentration of CK and CK-MB were lower than that after PCI operation (CK: 374 ± 580.15vs 4489 ± 2359.27, *p* < *0.01*; CK-MB: 39.89 ± 42.66 vs 344.23 ± 184.7, *p* < *0.01*). Additionally, the association between dsDNA, MPO/DNA and CK, CK-MB were explored, although there was no correlation been found, as well as dsDNA or MPO/DNA with enzymatic infarct size (creatine phosphokinase isoform MB area under the curve).

### Coronary dsDNA was more sensitive than other conventional prognostic markers

Receiver operating characteristic (ROC) curves were constructed to evaluate predictive value for hospital mortality (Fig. 4C). The area under the curve (AUC) of troponin T, CK-MB and dsDNA were 0.68 (95% CI 0.548 to 0.815), 0.57 (95% confidence interval (CI) 0.413 to 0.720), and 0.72 (95% CI: 0.612 to 0.881) respectively. The optimal cut-off value for troponin T was 0.17ug/L, for CK-MB 107.6U/L and for dsDNA 0.39ug/ml. The sensitivity and specificity for troponin T 61% and 28%, for CK-MB was 50% and 30%, for dsDNA 78% and 53%. Obviously coronary dsDNA on admission did reveal a higher AUC for the prediction of in-hospital MACEs.

## Discussion

To our knowledge, this is the first time that the relationship between neutrophils/NETs and STEMI has been studied in STEMI patients with TIMI 0 flow before emergency PCI. This study demonstrated 4 major findings: (1) neutrophils obtained from IRA release NETs (2) MPO/DNA and dsDNA (proposed as surrogate markers for NETs) and NETs-related factor TF to be highly increased in the IRA plasma compared with peripheral artery plasma (3) conventional antithrombotics such as heparin and tirofiban was ineffective to the NETs structures. (4) coronary dsDNA is independently associated with the development of in-hospital MACEs and more sensitive than other conventional prognostic markers in patients with STEMI undergoing primary PCI.

Circulating leukocytes, particularly monocytes, have been shown to play a central role in atherothrombosis^16,17^, neutrophils, the most abundant white blood cells, have not been investigated until recent years^18–23^. Accordingly, recent experimental evidence revealed the critical role of neutrophils/NETs in thrombotic events^24–26^ and mounting evidence implicates a potential role of NETs in linking sterile inflammation with thrombosis, including atherothrombosis^7,27^. Andreas Mangold has detected that NETs are found abundantly throughout coronary thrombi, serving as a primary scaffold for platelets and erythrocytes, as well as for fibrin^28^. Herein we observed that neutrophils isolated from IRA release NETs in STEMI patients (n = 36) as demonstrated by microscopy immunofluorescence. We also found, in agreement with the findings of others, that extracellular dsDNA levels and NET-specific marker MPO/DNA complexes are also elevated in plasma samples obtained from IRA. In addition, conventional antithrombotics such as heparin and tirofiban was ineffective to the NETs structures. In all, the evidence suggests that neutrophils/NETs are a possible key player in the pathogenesis of NET-related atherothrombosis. Simultaneously, our study patients who suffer from hospital adverse events had higher levels of dsDNA compared to patients who did not develop adverse events. Andreas Mangold observed that coronary blood dsDNA level was correlated positively with thrombus NET burden^28^, which suggests that circulating dsDNA primarily originates from NETs. Meanwhile, they showed that coronary NET burden correlated negatively with ST-segment resolution, which corresponds to the no-reflow phenomenon, consistently demonstrated to be a strong independent predictor of adverse clinical outcome. In the present study, we observed that increased levels of dsDNA from IRA are associated with the in-hospital MACEs among STEMI patients undergoing pPCI. Our study expanded the sample size, reconfirmed the previous studies conclusion and identified dsDNA as a potential therapeutic target for STEMI patients.

Additionally, most of the in-hospital complications were cardiogenic shock. The strongest factors related to cardiogenic shock were infarct size(IS) and residual LV function. Therefore, with the use of logistic-regression models, the others risk factors associated with IS and LV function were estimated as the predicted probability of the occurrence in-hospital adverse events. In a multivariate regression model, coronary dsDNA remained an independent correlation with in-hospital MACEs. In addition, we explored the association between the change of CK-MB and AUC CK-MB, which commonly used in evaluating the degree of myocardial necrosis in clinical practice and associated with adverse clinical outcomes^13–15^, and NETs (dsDNA, MPO/DNA). Although, there was no correction been found, coronary dsDNA was more sensitive than other conventional prognostic markers, such as troponin T and CK-MB. We speculated that these results associated with NET-related atherothrombosis and the mechanism should be explored more precisely in future studies.

Though there is significant evidence of a critical role for neutrophils/NETs in the atherosclerotic thrombotic process^18–20,23,29–31^, their impact on thrombogenesis was, until recently, questioned for acquiring or producing tissue factor (TF). Experimental data showed neutrophils/NETs acquire microparticle-derived TF while others suggested that TF is produced by neutrophils under inflammatory stimuli^32^. Osterud *et al*. reported that isolated neutrophils fail to produce TF when stimulated with lipopolysaccharide alone or in conjunction with phorbol myristate acetate(PMA)^33^. Simultaneously, another study demonstrated the expression of TF by neutrophils isolated from the bronchoalveolar fluid from patients with acute respiratory distress syndrome^34^. In relation to this, evidence from Dimitrios A. Stakos *et al*. showed that NETs express functional TF in the culprit artery of acute myocardial infarction^35^. For our study, in the perspective of concentration, we observed TF levels in the IRA were high compared with the levels in a radial artery. Whether elevated TF was associated with infarct-related arterial neutrophils/NETs or not, there was a need for further study and data in this area. Inflammatory factors such as TNF-α, which was thought to regulate neutrophils to release NETs and then generate TF^35^, was observed at higher levels in IRA than peripheral arteries, although this difference was not statistically significant.

## Limitations

This study has several limitations. First, only STEMI patients with TIMI 0 flow were included; therefore, the findings may not be generalizable to patients with TIMI > 0. Secondly, since the majority of our patients were successfully treated with pPCI (TIMI > 3; reperfusion success with pPCI influences the extent of myocardial damage^36^), further studies are necessary to demonstrate if our results are applicable to patients with unsuccessful PCI. Finally, our study was mostly descriptive, the mechanism should be explored more precisely in future studies. However, our data reconfirmed that the NETs are associated with STIM, and identified dsDNA as a potential therapeutic target for STEMI patients.

## Conclusion

Our study indicated that NETs were associated with STIM, and occurrence of in-hospital MACEs. Although our study was limited by a small sample, sensitivity analysis-bootstrap show that the results were robust.

## Methods and Materials

### Patients and samples

Between January and December 2017, one hundred and seventy consecutive patients with STEMI (78.9% men, mean age 59 ± 1years) admitted to the hospital within 12 hours of the onset of symptoms were prospectively enrolled in the present study. Patients undergoing Primary percutaneous coronary intervention (PCI) and fulfilling the following criteria were included in the study: (1) persistent severe chest pain >30 min, (2) ST-segment elevation of >1 mm in 2 consecutive leads on electrocardiogram, (3) a new occurrence of left bundle branch conduction block, or adjacent leads appear Q wave; myocardial enzymes or troponin increased, (4) TIMI flow 0 before PCI.

Patients with the following criteria were excluded: treatment with thrombolytic drugs in the previous 24 hours, cardiogenic shock in the previous 24 hours, active infection, previously diagnosed systemic inflammatory disease, known malignancy, end-stage liver or renal failure, under immunosuppression, recent trauma, and a refusal to enroll in the research study. All patients were given 300 mg of aspirin, 600 mg of clopidogrel and 30 mg atorvastatin. Informed consent was obtained from all patients.

In total, 83 patients were included in the present study, and this sample was able to detect the change corresponding to an odds ratio of 0.504 when coronary dsDNA is increased to one standard deviation above its median, assuming a baseline probability of 0.28 for the MACEs in our sample with 80% power at a 0.050 significance.

All PCI procedures were performed using the standard approach with a 6-Fr guiding catheter. After administration of intravenous heparin (100 IU/kg) and a loading dose of clopidogrel (300 mg), direct stenting was performed whenever possible; in the remaining cases, balloon pre-dilatation was performed. In patients treated with tirofiban, the agent was administered after the PCI procedure in the coronary care unit.

**Figure 5:**
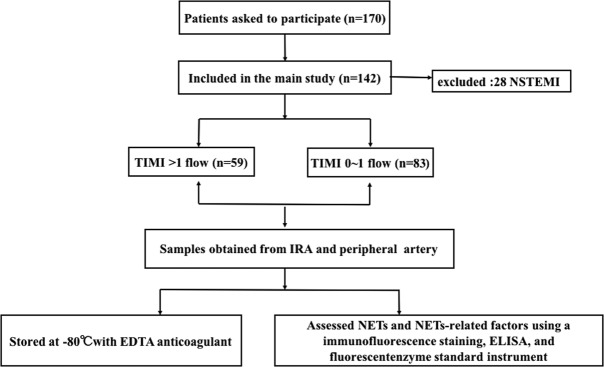
Flow chart.

For sample collection, a total of 10 to 20 mL of blood from the IRA was aspirated with a commercial thrombectomy catheter (Export [Medtronic, MN]). In parallel, radial blood was drawn from the radial artery. All blood samples were treated with ethylenediamnetetraacetic acid (EDTA) anticoagulant. A flow chart providing information related to recruitment and inclusion in the present study was presented in Fig. 5. This prospective cohort study was in compliance with the Declaration of Helsinki and was approved by the Ethics Committees of First Hospital of Xi’an Jiaotong University. All patients were given written informed consent.

### Detection of NETs structures and NETs-related factors

Neutrophils were isolated from human IRA or peripheral blood according to the manufacturer’s protocol (Human peripheral blood neutrophil cell isolation kits purchased from Haoyang Biotechnology Tianjin Beijing, China). The structure of NETs was detected using immunofluorescence and observed using confocal microscopy (TCS, Leica USA). SYTOX Green DNA was used to detect the dsDNA content in the plasma of IRA arteries or radial arteries. The MPO/DNA complexes was measured by ELISA^37,38^. At the same time, the concentrations of TF and TNF-α of coronary blood and radial blood were also measured.

### Definition of major adverse cardiac events

Cardiogenic shock: 1. Systolic blood pressure (SBP) ≤90 mmHg or mean arterial pressure drop ≥30 mmHg, or patients with hypertension decreased by 60 mmHg compared to the original systolic blood pressure for at least 30 minutes. 2. cardiac index (CI) ≤2.2 L/(min · m^2^). 3. organ hypoperfusion: changes in demeanor, cyanosis, limb chills, decreased urine output [<0.5 ml/kg · h]; Ventricular arrhythmias, ECG shows a wide QRS wave; Infarction recurrence: recurrence of myocardial infarction and elevated myoglobin; Heart failure, difficulty breathing, acute pulmonary edema, oliguria; Cardiac death: death within 24 H after admission.

### Statistical analyses

Power analysis and sample size was using to calculate the sample size. All analyses were performed using SPSS 21.0 v software. Continuous data were presented as mean ± SD, and were compared using Student t test. Categorical data were expressed as numbers and percentages. The relationship was examined using Spearman’s rank correlation test. A stepwise multivariate logistic regression analysis was performed to identify the independent risk factors of MACEs. The analyses included variables associated with the dependent variable in univariate analysis that did not have significant correlation between them. The clinical cut-off points were determined from the receiver operating characteristic curve. Statistical significance was set at *p* < *0.05*.
